# Twelve Tips for Utilizing Residency Program Social Media Accounts for Modified Residency Recruitment

**DOI:** 10.15694/mep.2020.000178.1

**Published:** 2020-08-26

**Authors:** Rakhee K. Bhayani, Laurel Fick, Dawn Dillman, Dink A. Jardine, Amy S. Oxentenko, Avital O'Glasser

**Affiliations:** 1 Washington University School of Medicine; 2 Ascension St. Vincent Hospital; 3 Oregon Health & Science University; 4 Navy Medicine Readiness and Training Command; 5 Mayo Clinic

**Keywords:** social media, Twitter, Facebook, Instagram, residency program, resident interviews, resident recruitment

## Abstract

This article was migrated. The article was marked as recommended.

Social media use across the health professions has significantly expanded in recent years. Specific attention has been paid to both the value of social media use in graduate medical education with residency program twitter accounts. More recently, social media has been examined for its role in supporting the rapid expansion of information exchange and connection across digital and virtual platforms during the COVID-19 pandemic. With the ongoing response to the pandemic, the 2020-2021 residency application cycle is anticipated to be a completely virtual interview process. Here, we draw from our collective experiences managing, maturing, and maximizing social media accounts for residency programs and GME to provide practical tips for using social media for the upcoming virtual interview season.

## Introduction

As the global impact of the novel coronavirus of 2019 (COVID-19) spread to the United States in the early months of 2020, the impact on health care systems as well as undergraduate and graduate medical education (UME and GME) unfolded swiftly and dramatically. Classroom-based education and clinical experiences were transformed overnight ( Ferrel and Ryan, 2020). As we entered May 2020, the strong likelihood of a much longer period of disruption became increasingly apparent. With the precedent already set in the antecedent months for work-related travel bans and cancellation or virtual modifications to in-person meetings, on May 7th, 2020, the American Association of Medical Colleges (AAMC) announced that it strongly recommended that 2020-2021 residency interviews be held virtually ( American Association of Medical Colleges, 2020a).

In-person residency interviews are a long-standing focus of the academic cycle. Significant student investment, including time away from rotations for travel and personal out-of-pocket expenses, is spent during the last year of medical school ( Benson, Stickle, and Raszka, 2015; Walling *et al.*, 2017; Fogel *et al.*, 2018). International Medical Graduates (IMGs) and other non-traditional applicants face additional challenges ( Leon *et al.*, 2007; Desbiens and Vidaillet, 2010; Chen *et al.*, 2011). The number of applications has increased ( American Association of Medical Colleges, 2020b), and programs must balance bandwidth for the number of applications they can review, the number of interview spots available, and the ultimate number of resident positions to rank.

The AAMC’s announcement invited logical comparisons between the intersections of web-based resources, social media, and virtual interviews within other sectors including business and higher education ( American Association of Medical Colleges, 2020a; Carpentier *et al.*, 2017; Carpentier, Van Hoye and Weijters, 2019; Carpentier, Van Hoye and Weng, 2019; Rutter, Roper and Lettice, 2016). The announcement immediately created the question for the GME community of how to replicate the on-campus interview experience virtually. While the application and interview process has clearly identified stressors, it also facilitates the ability for applicants to gain first-hand, in-person experience with programs to empower applicants’ rank list decisions. More than simply sharing program specifics or offering campus tours, the interview day also affords applicants the chance to gauge the fit and feel of a program and assess program culture. While certain elements of an interview day might lend themselves readily to a virtual equivalent, could program “culture” or “brand” be as easily transmissible? Literature prior to the onset of the COVID-19 pandemic provided early guidance about effectiveness versus limitations of such modalities ( Vadi *et al.*, 2016; Pourmand *et al.*, 2018).

Social media use by residency programs has expanded in recent years, with a growing body of literature analyzing its use. Reasons for use include education (both locally and on a broader/global level), dissemination of program and resident accomplishments, and resident engagement ( O’Glasser, Desai and Cooney, 2019; Scott *et al.*, 2014; Bergl, Narang and Arora, 2015; Galiatsatos *et al.*, 2016; Haas *et al.*, 2016; Diller and Yarris, 2017; Sterling *et al.*, 2017; Xie *et al.,* 2018; Fick, Potini and Axon, 2019; St Claire *et al.*, 2019; Azoury *et al.*, 2020). There is also a small subset of literature that specifically explores the intersection between social media accounts and recruitment across specialties, in addition to more traditional web-based resources ( Embi, Desai and Cooney, 2003; Schweitzer, Hannan and Coren, 2012; Go, Klaassen and Chamberlain, 2012b, 2012a; Golden *et al.*, 2012; Deloney *et al.*, 2014; George *et al.,* 2014; McHugh *et al.*, 2014; Pillow *et al.*, 2014; Sterling *et al.*, 2017; Renew *et al.*, 2019; Matchett, Astor and Maursetter, 2020). More recently, social media channels have been noted to be able to specifically convey residency program culture and other intangibles ( O’Glasser, Desai and Cooney, 2019). Today’s applicants are nearly all members of the millennial generation who may be uniquely suited to rapidly adapt to utilizing social media to learn about residency programs ( Fick, Potini and Axon, 2019).

We strongly believe that utilizing and optimizing residency program social media accounts will be a significant component of a modified residency program recruitment season during the 2020-2021 academic year. Social media alone will not supplant other traditional and non-traditional elements of the interview and recruitment process (emails, websites, video interviews, information sessions, campus tours), but here we offer our twelve tips for including a social media presence as an integral part of the process/innovations of researching programs, interviewing, and finalizing match lists.

## Twelve Tips

### Tip 1: Get started with your preferred social media platform

All social media platforms are not created equally. The reach, demographics, and engagement statics vary between platforms and can change rapidly over time. Your choice of platform should consider you and your team’s current comfort level with the platform(s), the goal of your outreach strategy, and the audience you are trying to reach ( Kind *et al.*, 2014). Be deliberate about which platform you choose and include in your development team a trainee to ensure you are reaching the audience you intend to reach, or poll your program in advance regarding preferred platforms. Twitter has historically been the most used platform for healthcare professional communication ( Raghupathi and Raghupathi, 2014) and has a mature medical education-focused community. The authors primarily use Twitter, followed by Instagram, for engagement and creating a community for purposes of recruitment through a cross-platform presence where resources allow may be the most effective solution. A brief summary of selected benefits and drawbacks of each platform is provided ( Table 1) ( Raghupathi and Raghupathi, 2014; Perrin and Anderson, 2019; Kullar *et al*, 2020; Zote, 2020).

Once your platform or platforms have been chosen, research and review other similar accounts on that platform. Images draw the eye to Twitter and Facebook posts and are the backbone of Instagram and Snapchat posts. Videos form the basis for all TikTok postings and can be shared across most other platforms. All platforms use some variation of hashtags, which are created by placing a “#” in front of a word or short phrase without punctuation, such as #MedTwitter or #MedEd, to track similar content over accounts. Platforms also index these hashtags for search functions, and users can search on a hashtag for new related content. Tagging, as denoted by the “@” symbol, is done by placing a username on that platform in the post or linked to the post, and can increase your readership by distributing content over a more extensive audience pool; however, it can also be seen as intrusive, so use it with caution. A few hours spent researching your chosen platform can inform your decisions for later postings and maximize engagement.

**Table 1.** Comparison of Selected Social Media Platforms.

Platform	Pro	Con	Comments
Facebook (FB)	Largest audience. Familiar to most users and reaches nearly 7 in 10 U.S. adults. Can post live video for immediate engagement. Easily cross post with IG.	Older audience demographic.	Not used as routinely among young adults for day to day activities but rather for answering specific questions and contact information sharing.
Instagram (IG)	Growing audience and near parity with FB in young adult demographic. Highest individual post engagement of all platforms. Easily cross posts with FB.	Photos and videos drive followers. Heavily used by commercial marketing and has frequent paid ads in feeds.	Considered a growth area by marketing professionals for young adult demographic. Along with Twitter can develop more longitudinal engagement (daily or near daily).
Twitter	Robust medical education community. Historically, the most popular social media platform for healthcare communication. Considered the top platform to discover new ideas, organizations, or brands. Has respected educational uses including TweetChats and Tweetorials.	Smaller overall and daily user audience.	Mature medical education community and growing research area including scholarly work for best practices. Can be used for longitudinal engagement.
LinkedIn	Largest professional and Business-to-Business site. Used by professionals for job search activities.	Small daily engagement.	Limited daily engagement to share and build culture but having a presence is likely helpful.

( Raghupathi and Raghupathi, 2014; Perrin and Anderson, 2019; Kullar *et al*, 2020; Zote, 2020)

### Tip 2: Get started at the program level

Before beginning your social media journey, familiarize yourself with the social media policies and procedures outlined by your institution. You may need permission to create an official account in order to utilize the existing branding material. Some institutions require a media release form for any photos of non-employees that you are planning to post. It’s best to be aware of these expectations at the outset. In addition to institution specific guidelines, always keep in mind the more universal Health Insurance Portability and Accountability Act (HIPAA) guidelines if posts include computer screens with potential patient information, patients in the background, or medical case presentations.

As you create your social media team, determine who will be optimal to compile content for the posts, devote adequate time to engage with the audience, and deliver consistent tone, language and messaging that reflects the goals of the residency program. Some programs have chief residents, staff members, associate program directors, or some combination assigned to manage the account. Regularly curating information for posts that can be drafted and scheduled to automatically post can help keep up with the demand of your social media account. More timely posts can be interspersed between the scheduled posts.

Start by following other medical schools, GME programs, medical societies, alumni from your program, current faculty, leaders in the GME community, and prominent journals that are specific to your specialty. If you are new to social media, you can glean ideas from other accounts that can help guide you. Never lose sight of the audience and your mission-based goal of highlighting all that your program has to offer. Over time, you will gain followers and increase the engagement of potential applicants. Now, you are ready to create a cohesive strategy for your program’s social media presence.

### Tip 3: Set boundaries

Social media platforms are integrated into our daily lives and have become a mainstream way to consume information. With its widespread use, it is important to set boundaries that will be followed in a consistent manner by your social media team. While you hope to increase your visibility to the potential applicant pool and increase engagement, it is best to avoid following the accounts of potential applicants. Having applicants follow you, like posts, and comment on social media may be deemed as demonstrated interest in your program. However, it is important that you do not give an unfair advantage to those that engage directly with your account on social media. This would penalize applicants who may not be active on social media or those that are reading posts and not engaging with them. Many applicants may be cautious about who they are engaging publicly on social media given there are many competing programs on the various platforms, and such engagement may force them to disclose a preference for one program over another. If you do interact with those that leave comments, ensure that your level of engagement is uniform across the board. Having clear and consistent boundaries in how you interact with your audience should be communicated to your social media team.

### Tip 4: Don’t get narrow-sighted and only post “current events” or brand new accomplishments

**Figure 1.**
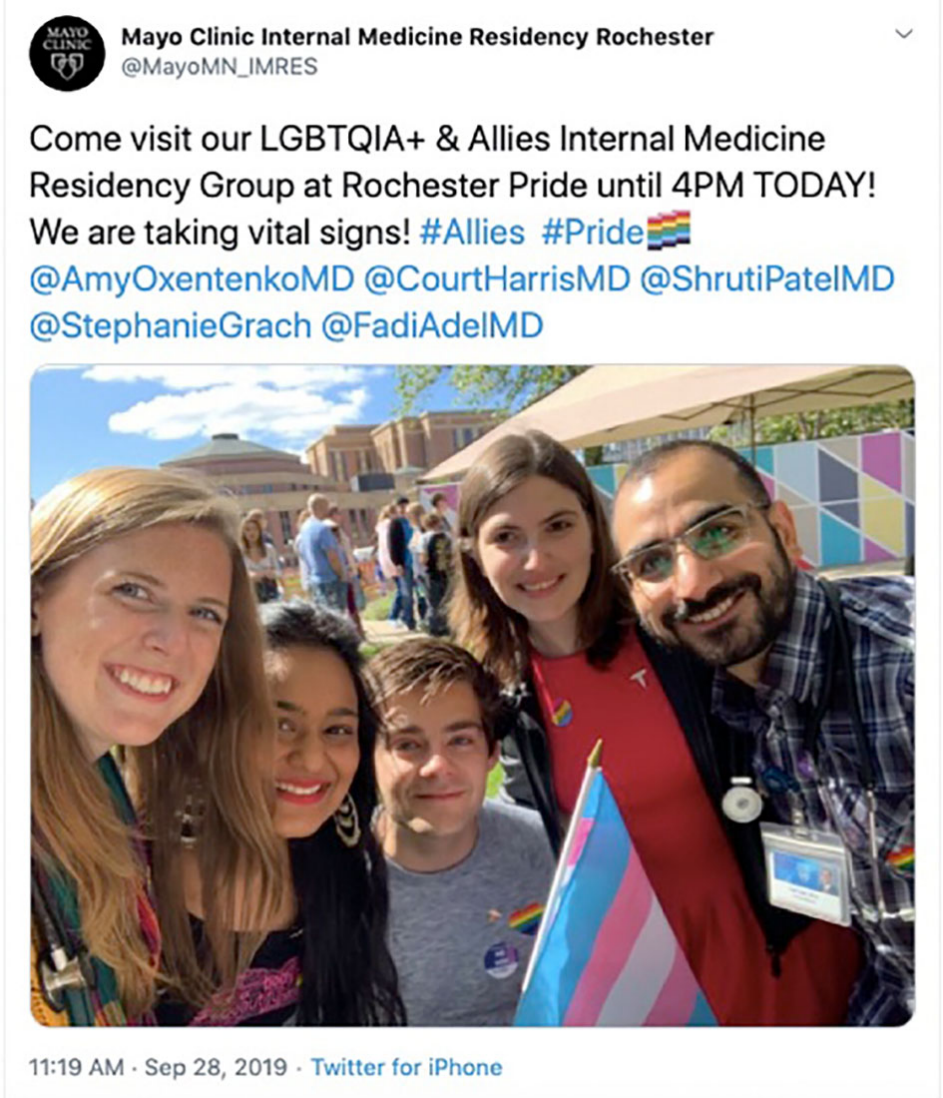
Example of how an internal medicine residency Twitter account uses a Tweet to highlight curricular goals, the presence of an interest group, and community volunteerism within the program. Available at https://twitter.com/MayoMN_IMRES/status/1178011419138121728?s=20

**Figure 2.**
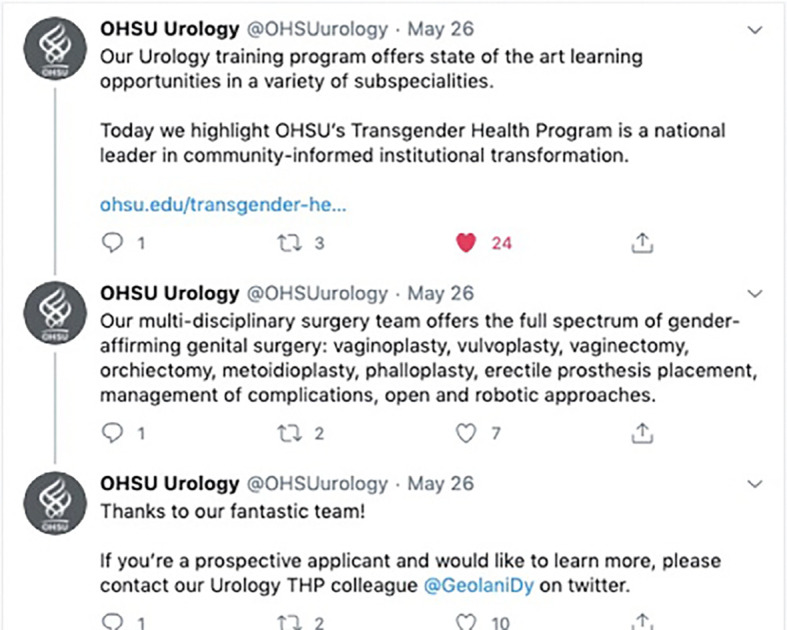
Example of how a surgical specialty Twitter account uses a series of three tweets to highlight curricular content and training opportunities as part of an institutional multidisciplinary care team. Available at: https://twitter.com/OHSUurology/status/1265286832897454080?s=20; https://twitter.com/OHSUurology/status/1265286834872967168?s=20; https://twitter.com/OHSUurology/status/1265286836550696962?s=20

It is easy to create social media posts that highlight current events going on in the training program and its immediate community. However, only relying on new accomplishments will have limitations, especially with social distancing and limitations on activities. What if there are no new accomplishments thus creating a long void of content? Do the current accomplishments truly capture the entirety of a program’s notable features? Although it is wonderful to capture trainee- and program-related achievements as they happen, be sure to work in regular posts regarding the program itself and curricular highlights. For example, if a program has global health opportunities, these could be showcased alongside pictures of trainees from past overseas experiences. In the setting of the COVID19 pandemic and limited travel, a program would miss out on highlighting such a program feature if it only relied on current events. A program should think about the following: What are the special features of our curriculum? What experiences do we have that others may not? What are things that trainees are specifically looking for as differentiators? For example, if your program has an “LGBTQIA & Allies” interest group or Women in Medicine programming, showcasing these initiatives and related curricular content may send the message to prospective applicants that the program is welcoming and inclusive ( Figures 1 and 2). As a program reviews its core values, these should be clearly reflected in the esprit de corps of the social media posts. When posting trainee accomplishments (e.g., publications, presentations, awards, etc.), a program should be conscientious of representation by all groups in the program. Another way to develop content for posts would be to look at the program’s calendar of events, and disseminate pictures and content from such activities, such as program retreats, workshops, social events and skill-building experiences.

### Tip 5: Get outside the walls of work

**Figure 3.**
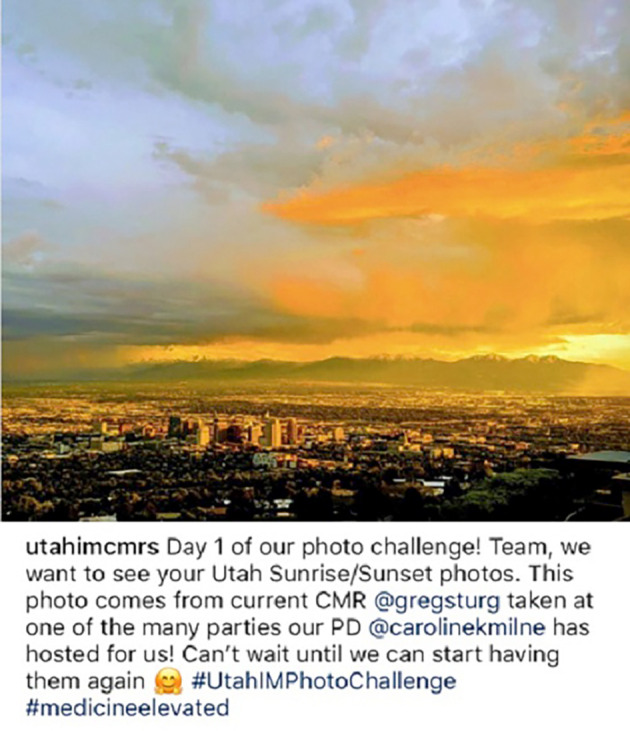
Example of an internal medicine residency program Instagram account utilizing social media to share information about the beautiful landscapes around their hospital, as well as a brief description of Program Director led traditions: Available: https://www.instagram.com/p/CADL2d4hZE6/?igshid=rkyytyp9wv9x

Applicants to a program will want to find out all they can about the program, but they will also want to know what it is like outside the walls of the hospital and clinic, both on campus and around the city. The “fit and feel” that trainees note when visiting a program is informed by the people they meet and experiences in the local area before and after the interview day. With virtual interviews, programs will need to be particularly mindful to capture those things that make the campus and city appealing, since applicants will need to live vicariously through social media posts to capture this essence. Programs should inform their current trainees to take pictures of social events outside of work, not only capturing group activities, but also showcasing hobbies and activities that individuals engage in. What do trainees do on days off? What are local activities that trainees enjoy? Attention to this level of detail will allow prospective trainees to look at a program and see themselves there. Such highlights may include fitness activities, farmers’ markets, outdoor adventures, local events, favorite restaurants or food trucks, and much more. Any trainee-provided photos should be reviewed by program administration prior to posting on behalf of the program to assess for appropriateness and to ensure privacy laws were not violated. Do not forget to showcase examples of where trainees live. If they can afford to purchase a house or condo, that could be highlighted. Be sure to highlight the landscape and include cityscape photos, which will allow a program to paint the visual picture for applicants of the city they will live and work in and ultimately call “home”--very much consistent with the “love where you work and the work you do there” theme within #medtwitter ( O’Glasser, Desai and Cooney, 2019) ( Figure 3).

### Tip 6: Be intentional and proactive with diversity, equity, and inclusion

**Figure 4.**
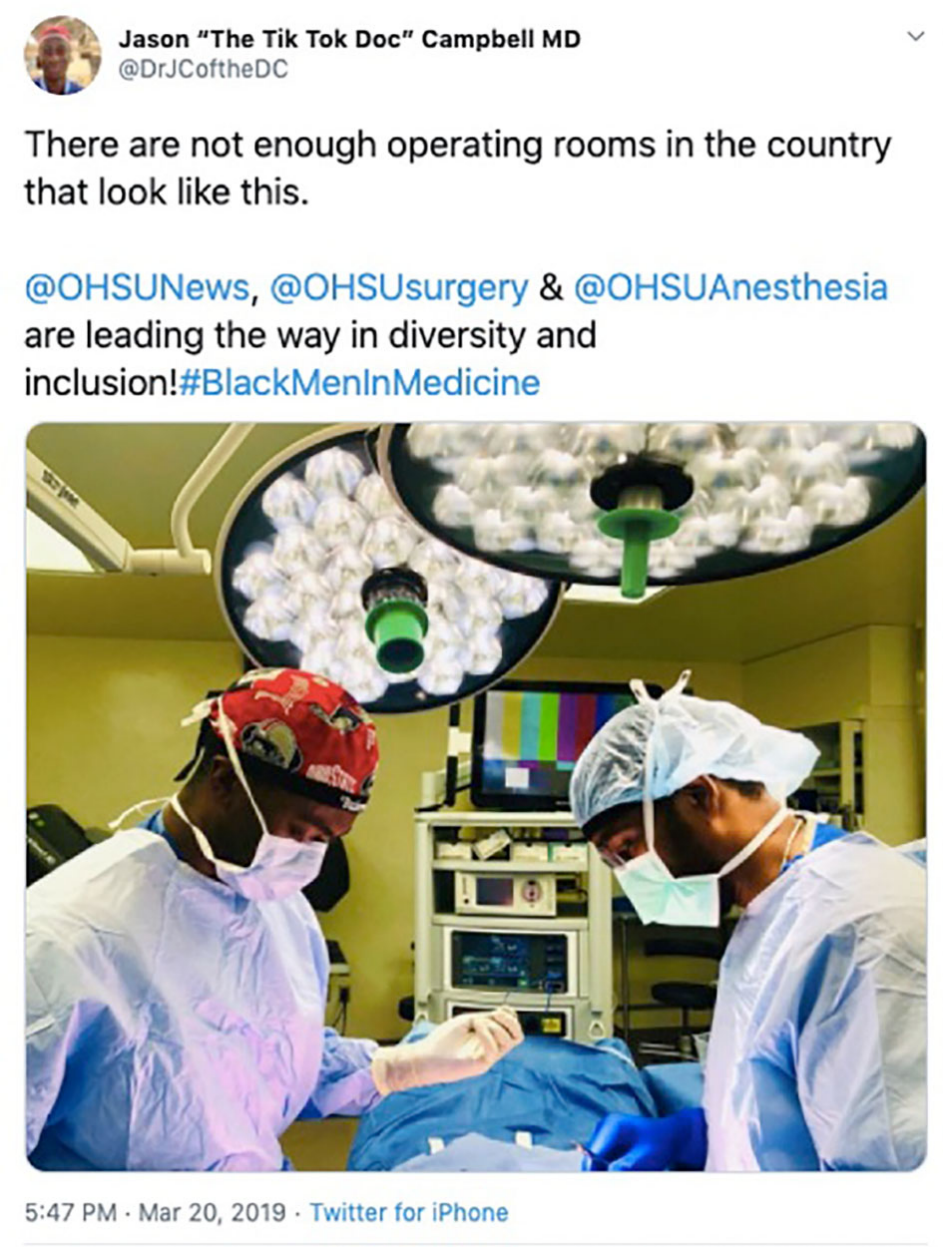
An anesthesiology resident (Dr. Jason Campbell, left) sharing his experience working in a perioperative training setting that fosters diversity and inclusion (seen with Dr. Don Spight, right). Available: https://twitter.com/DrJCoftheDC/status/1108530571192360961?s=20

One of the features that any applicant to a program is attempting to assess is “will I be supported at this program?” This assessment becomes particularly difficult when there are no in-person interactions during the interview day to see off-hand interactions with staff or patients that might give insight into the day-to-day culture. It then becomes incumbent on programs to demonstrate that applicants who are persons of color, LGBTQIA, or identify with another under-represented group are welcome and would be successful in the program. This is best done as an intentional, multi-pronged approach. First, be intentional about systematically evaluating who is being promoted on social media, and attempt to include all members of the program. This includes cultivating posts to include different postgraduate years. Consider how you might incorporate each trainee and their experience deliberately. This has the beneficial effect of not only working to help recruitment, but also supporting the trainees already in the program. Second, include pictures, and attempt to represent visible diversity if possible ( Figure 4). Third, call out diversity support and sponsorship when applicable. If there are events your program is doing to improve the pipeline into college or medical school, or collaborations with on-campus groups, share information about those. Also post about specifics of mentorship or development opportunities for trainees **.**

### Tip 7: Share trainee and program “wins” and accomplishments

**Figure 5.**
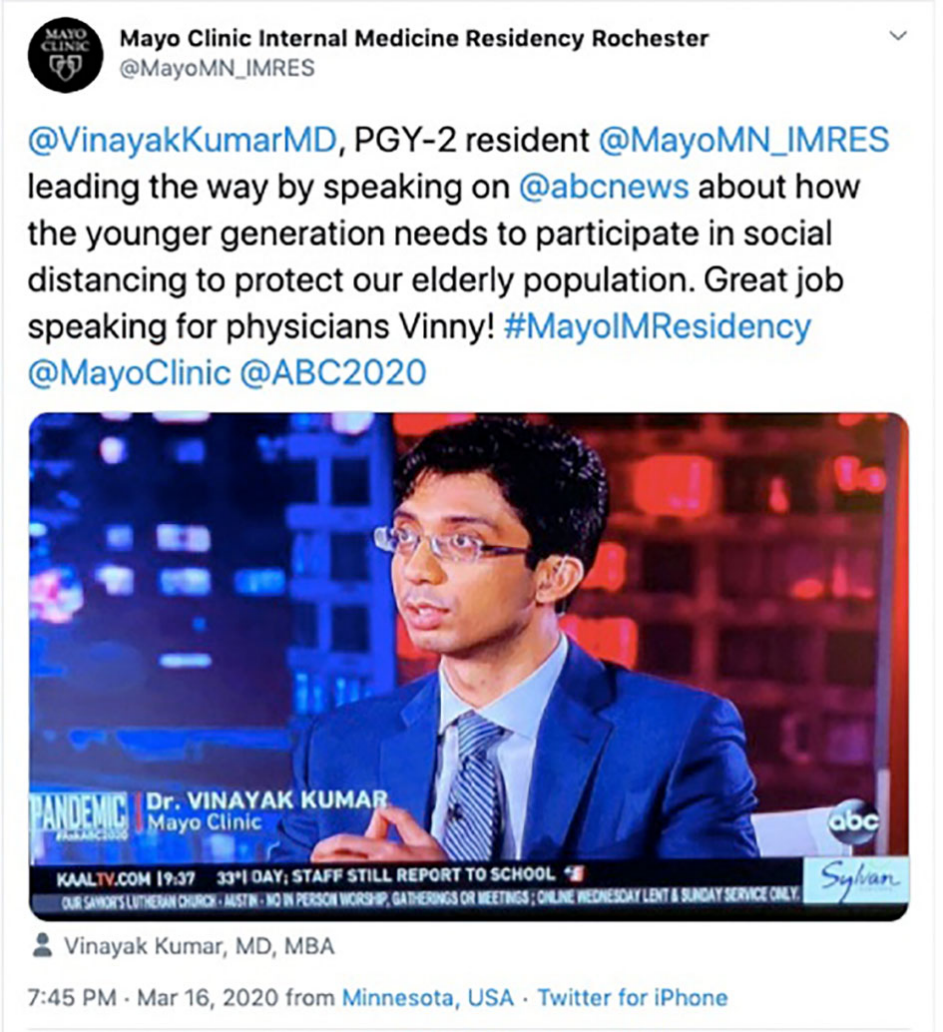
An internal medicine residency program highlighting unique advocacy and public education endeavors by a trainee. Available at: https://twitter.com/MayoMN_IMRES/status/1239744577030049794?s=20

Sharing your program’s culture and community necessitates sharing what your organization values. Posting or reposting moments that highlight current trainee or alumni accomplishments is a powerful way to do this. While research publications and presentations are frequently used and outstanding opportunities to highlight scholarship (representative hashtags include #ScholarlySunday used by @MayoMN_IMRES and #OHSUscholarship used by @OHSUIMRes), consider other accomplishments that highlight other program values including non-traditional scholarly projects and presentation opportunities ( Figure 5). Leadership positions in national and professional societies reflect the program’s professional and leadership development of its trainees. Highlighting community volunteering by residents or organized community engagement activities promote your program’s culture of volunteerism. Posting of legislative or public policy involvement activities highlight the importance of advocacy in your program. Look for opportunities to share these moments that make you program to lead your program and share those. Thus express the culture of the program while simultaneously amplifying your trainee’s accomplishments.

### Tip 8: Conveylearning culture and #meded innovations

Programs should already be utilizing social media to celebrate the academic accomplishments of residents and faculty by posting pictures of presentations, posters, visual abstracts, and links to oral presentations or manuscripts with tags for the physician(s) and sponsoring conference or journal. To demonstrate a program’s unique culture of curiosity, showcase virtual learning conferences, novel teaching techniques, and creative approaches to modern learning like interactive Twitter question and answer sessions.

Invite applicants to participate in virtual conferences as able using formats such as Facebook Live or Instagram Live. Programs that utilize simulation should show pictures and videos of learners in action. Feature interesting cases from your program using HIPAA-compliant imaging to demonstrate the diverse pathology your learners encounter. Upload teaching points from live conferences across your program platforms to show dedication to asynchronous learning for off-campus, or nightshift residents. “Conversations” between the program account, residents, and faculty can also help convey learning culture through their tone and content.

### Tip 9: Establish a local community within your program and department

Train current trainees and faculty in the value of social media to their career development, as well as how to create an effective post. The more individuals are posting and tagging the program account, the more easily the program feed will be able to draw from a diverse group of voices and more stay active with up to date achievements. In the program feed, consider highlighting the accomplishments of faculty if the faculty will be working with residents. There may be an applicant who is looking to work with a mentor on a specific project, and this may be a way of letting that person know they would be supported at your program. Frequently, the “conversations” between trainees and faculty (as well as fellows) that unfold via social media posts can be a means of insight into the culture and unique training elements of a program for applicants as well--what topics of interest are discussed (e.g. addiction medicine, point of care ultrasound, etc.) and how might psychological safety and respect for learners be conveyed in a few hundred characters. Graduates who continue to be involved on social media may function as benchmarks for achievement and provide context for culture as well.

### Tip 10: Establish a local community outside your specialty at your institution

As noted in Tip 9, social media is capable of conveying the relationships between a program and its residents as well as between members of a clinical department, its embedded divisions, faculty, and fellows. Additionally, relationships exist beyond the “boundaries” of the residency program itself and its affiliated clinical department. Social media can help build and convey relationships between a residency program and its affiliated department and other residency programs and their departments within a hospital. These conversations can also be essential for communicating core values and aspects of a training program.

**Figure 6.**
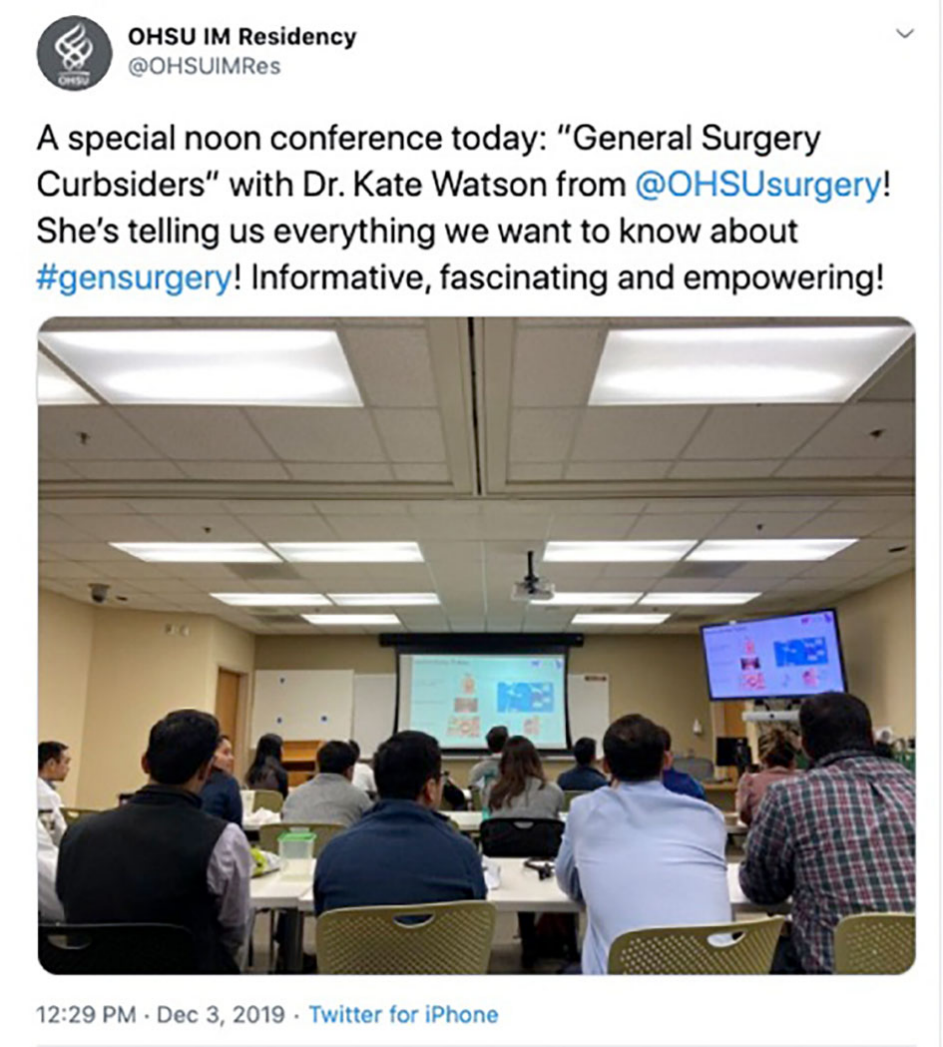
An internal medicine residency program highlighting a unique collaboration, with a general surgery resident delivering a multidisciplinary resident teaching conference. Available at: https://twitter.com/OHSUIMRes/status/1201961648774242304?s=20

Departments frequently overlap in providing clinical care, and social media posts can convey specifics of the clinical training environment and curricular highlights. Content of social media posts can also highlight overlap and innovations in the classroom or lecture-based setting ( Figure 6). The tone and phrasing of these posts can capture and convey the culture and core values of residency programs in terms of valuing multidisciplinary collaboration, fostering mutual respect, and minimizing hierarchies and silos within medicine.

**Figure 7.**
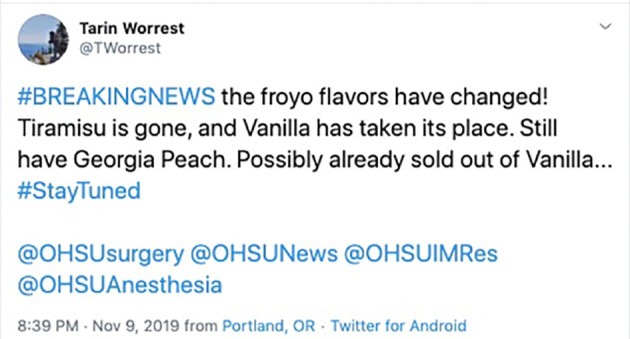
General surgery resident Dr. Tarin Worrest provides Twitter-based updated about the hospital cafeteria’s froyo flavors, which are anticipated by OHSU residents and faculty across specialties. Her tweets are often tagged with the hashtag #OHSUFroyoReport. Available at: https://twitter.com/TWorrest/status/1193387579825016832?s=20

Additionally, multiple social media accounts can convey elements of program culture important to applicants through “social” or more casual posts. Informal tone and friendly banter can easily maintain collegiality and not risk becoming unprofessional. For example, tweets may share off-campus work/life balance or non-clinical specifics of campus offerings ( Figure 7). Programs may also more deliberately overlap in their recruitment related content.

For programs at institutions without additional GME training programs, we recommend maintaining broader criteria for multidisciplinary partners who might have social media presences. Do residents or medical students from programs at other institutions rotate at your clinical site and overlap with your residents? Is there a nursing training program at your hospital? Do other ancillary services, such as physical or occupational therapy, have a social media account?

### Tip 11: Share “day in the life” features

**Figure 8.**
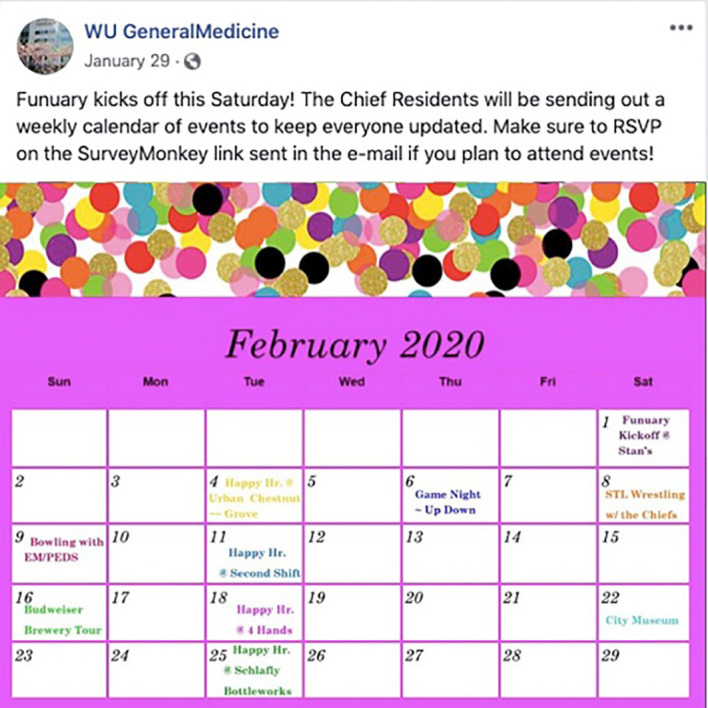
Example of how an internal medicine residency program uses Facebook to share a special schedule for the month of February Available at: https://www.facebook.com/100000393062664/posts/3032782253411514/?d=n

The in-person interview day, with the chance to observe resident didactic conferences and rounds in addition to a campus tour or program director presentations, affords applicants a chance to experience granular aspects of the day to day of a program. Social media can also be utilized throughout the annual cycle, rather than compressed into one day, to share the day-to-day and week-to-week elements of a program ( Figure 8). Utilize photographs and videos to add to the narrative of the residency experience-share candid moments in team rooms, conferences, and clinic spaces, though be sure to avoid any potential HIPAA violations if identifiable information is visible in the background (ex. Radiographs, patient list on a team room whiteboard, computer screens, etc.). Such posts have the ability to share long-standing community building residency program traditions that also highlight culture and values (for example, Saturday morning brunch after a week of night float), as well as the ability to “have fun.”

### Tip 12: Get really novel and creative!

**Figure 9.**
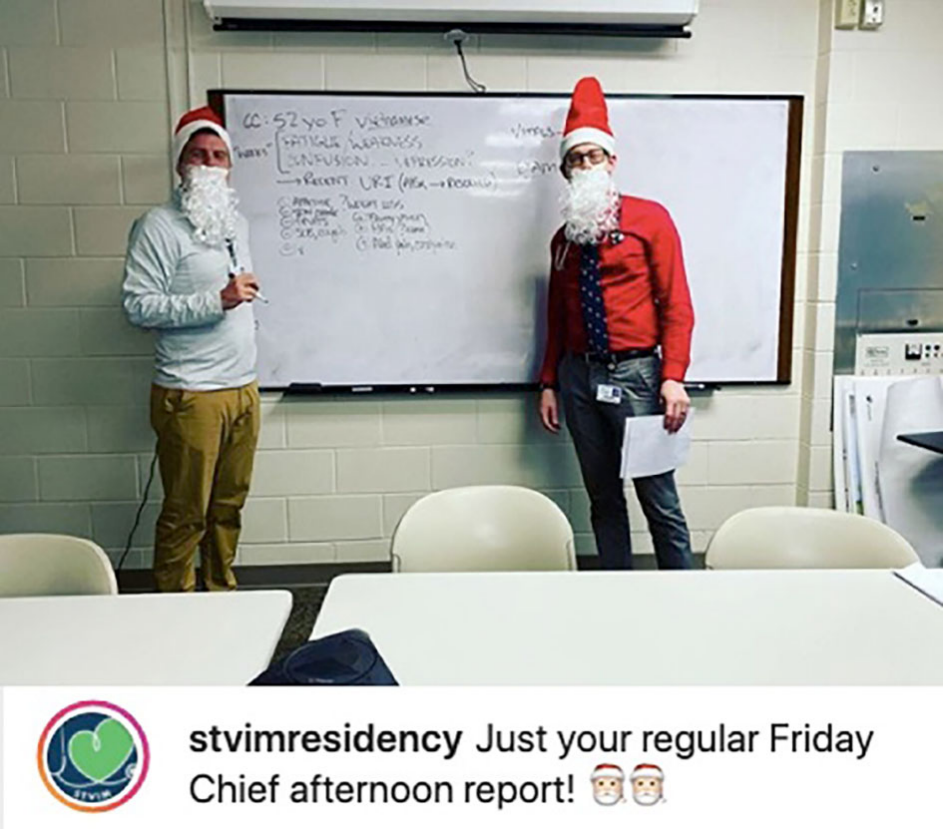
Chief Residents adding brevity and seasonal cheer to a weekly teaching conference. Available at: https://www.instagram.com/p/B6TqynRBR7-/?utm_source=ig_web_copy_link

Commit time and resources to explore new platforms (e.g. TikTok) or features within existing social media platforms (Instagram, Facebook, or Twitter “Stories” or “Live” functions) to continually appeal to the new generation of learners. Some programs may consider pooling resources to hire social media content consultants to manage or support this aspect of program advertisement. Consider utilizing stock photos and/or third-party apps such as WordSwag© to create aesthetically interesting posts. Drive engagement by tagging featured residents, affiliated medical schools, and professional societies, and by utilizing well-known, high yield (e.g. #residencylife, #anesthesiaresident) and program-specific hashtags (#stvimwellness, #washupeds, #OHSUscholarship, #ScholarlySunday). Geotags (tagging the post- or program location) can also help applicants discover previously unknown programs in specific geographies of interest. Show your program’s personality with a mix of educational and fun posts such as costume contests, wellness program activities, resident spotlights, celebrating engagements and babies, honoring pandemic heroes, dissemination of program announcements, and resident talents ( Figure 9).

## Conclusion

Social media use by residency programs continues to evolve and become more integrated into the GME landscape. The experiences of these authors are that using social media for residency programs is feasible as well as rewarding and fulfilling, especially in terms of sharing successes of its trainees and highlights of program features. With the dramatic shift in graduate medical education, including the upcoming residency program recruitment and interview season, due to the COVID-19 pandemic, we anticipate that digital and virtual platforms like social media will continue to be an integral part of the GME landscape. Based on our reflections on the positive aspects of social media use for residency programs, we believe that social media can be used very intentionally and productively with the call for virtual residency program interviews. These twelve tips are shared to facilitate the crafting of dedicated social media strategies for the upcoming, and future, application and recruitment cycle.

## Take Home Messages

• Multiple social media platforms are utilized by residency programs, and the choice of specific platform can depend on local comfort and expertise.
• Twitter permits residency programs to share core values by sharing program structure and curricular features.
• Twitter affords residency programs the chance to share their joy about resident accomplishments.
• Social media can be used intentionally to support diversity, equity, and inclusion.
• The benefit of social media for recruitment also stems from the ability of applicants to witness “conversations” with faculty and residents.

## Notes On Contributors

Rakhee K. Bhayani, MD (@RakheeBhayaniMD) is an Associate Professor of Medicine in the Division of General Medicine at Washington University School of Medicine in St. Louis, Missouri. She is the Internal Medicine Residency Wellness director and is the founder and director of the Forum for Women in Medicine, a professional development initiative for women trainees. She is the account manager and creator for the Forum for Women in Medicine Facebook page and Twitter @WashUFWIM. ORCID: https://orcid.org/0000-0002-3646-9690

Laurel Fick, MD, FACP (@laurelfick) is an Associate Program Director, St. Vincent Internal Medicine Residency and Program Director of the Transitional Year Residency. Her major areas of interest are physician wellbeing and retention, mentorship, and medical education research ***.*** *She is an account manager/creator for @stvimresidency Instagram account.* ORCID: https://orcid.org/0000-0003-1115-2627

Dawn Dillman, MD (@DawnDillman1) is a Professor and Vice-Chair of Education for the Department of Anesthesiology & Perioperative Medicine, Oregon health & Science University, Portland, OR. She is a content creator for the @OHSUanesthesia Twitter account for the OHSU Department of Anesthesiology and Perioperative Medicine. ORCID: https://orcid.org/0000-0002-4059-534X

Dink Jardine, MD, FACS (@dinkjardine) is the Associated Designated Institutional Official and Director for Faculty Development for Navy Medicine Readiness and Training Command, Portsmouth, VA and an Assistant Professor in Surgery and Pediatrics at the Uniformed Services University. She is creating institutional standards for local programs to create social media branding to share their unique culture. ORCID: https://orcid.org/0000-0002-3634-7060

Amy Oxentenko, MD, FACP, FACG, AGAF (@AmyOxentenkoMD) is a Professor of Medicine and has served as the Program Director and Associate Chair for Residency Education for the Department of Medicine for Mayo Clinic, Rochester. She is transitioning to the role of Chair of Medicine for Mayo Clinic, Arizona. She has been one of the administrators for the @MayoMN_IMRES Twitter account for the Mayo Clinic Internal Medicine Residency Program. ORCID: https://orcid.org/0000-0002-5371-0968

Avital O’Glasser, MD, FACP, FHM (@aoglasser) is a hospitalist and Associate Professor of Medicine in the Division of Hospital Medicine, Department of Medicine and the Assistant Program Director for Social Media and Scholarship of the Internal Medicine Residency Program, Oregon Health & Science University, Portland, Oregon. She is an account creator and manager with the @OHSUIMRes Twitter account for the OHSU Internal Medicine Residency Program. ORCID: https://orcid.org/0000-0002-0223-405X

### Disclaimer

The views expressed in this presentation are those of the authors and do not necessarily reflect the official policy or position of the Department of the Navy, Department of Defense, or the United States Government.

Dr. Jardine a military service member. This work was prepared as part of my official duties. Title 17 U.S.C. 105 provides that “Copyright protection under this title is not available for any work of the United States Government.” Title 17 U.S.C. 101 defines a United States Government work as a work prepared by a military service member or employee of the United States Government as part of that person’s official duties.
