# Supplementary material for: Twelve Tips for Utilizing Residency Program Social Media Accounts for Modified Residency Recruitment
Source: MedEdPublish (2016). 2020 Aug 26;9:178. [Version 1] doi: 10.15694/mep.2020.000178.1 (PMC10967688; doi:10.15694/mep.2020.000178.1)
Supplement: Peer review history

# Reviewer report: Reviewer response for version 1

**DOI:** 10.21956/mep.20135.r31086

**Published:** 2021-02-25

**Authors:** Subha Ramani

**Affiliations:** 1 Harvard Medical School, Brigham and Women's Hospital

This review has been migrated. The reviewer awarded 4 stars out of 5 I enjoyed reading this article about using social media to convey the culture of an institution during the era of virtual residency interviews. Many of these strategies will be useful post-pandemic even when in-person interviews return. The authors provide a good introduction to set the stage for the tips and provide a useful overview to institutional use of social media.The paper is well written, well referenced and will be useful to all health professions educators involved in recruitment and selection of trainees. The focus on diversity, equity and inclusivity is critical.I have comments on a few of the tips:Tip 1- Here I see that the authors are trying to inform readers of the several platforms and their pros and cons. This tip could have been more organized as I feel like I am going back and forth a little. The procedures for each platform could get a little confusing especially for novice users. The table, on the other hand, is useful.Tip 2- This tip is very important, but I wonder whether team composition and role assignments are a separate tip from getting started at the program level.Tip 3- I agree completely with setting boundaries and would also recommend a clear disclaimer that emphasizes that those who do not engage are not at an unfair disadvantage. Communication among the team is important, but it is also important to communicate to the audience.Overall, the authors have emphasized how important it is to focus on the academic and non-academic aspects of the program culture including outside the walls of the institution. The last thing I would like to add is that programs should also find other ways to send the message about their culture for those individuals who are hesitant to engage in social media. There should not be a pressure to engage for anyone who is not comfortable.As the other reviewer has recommended, instructions and guidelines for the audience would be very important.

Reviewer Expertise:

NA

No decision status is available

# Reviewer report: Reviewer response for version 1

**DOI:** 10.21956/mep.20135.r31087

**Published:** 2020-09-30

**Authors:** Ken Masters

**Affiliations:** 1 Sultan Qaboos University

This review has been migrated. The reviewer awarded 4 stars out of 5 An interesting paper detailing the use of Residency Program social media accounts for modified residency recruitment. The paper begins clearly, by explaining the problem of disrupted face-to-face residency recruitment, brought about by COVID-19, and then offers tips on how to use social media to assist in the recruitment process.One of the problems with social media, identified in the paper, is that they can change over time – this problem is brought home by the recent turmoil over TikTok (which, in fairness to the authors, occurred after this paper had been published).I am surprised, though, that the authors do not mention Reddit. Although it would probably be more difficult to set up a viable type site (sub-reddit) with “followers”, Reddit would be a useful sounding board to gauge prospective residents’ attitudes on a host of issues. It would take careful work, but, in the right hands, and used judiciously, could bear fruit.Also, I think the authors might like to highlight a point they make, but somewhat bury, in the last tip: “Some programs may consider pooling resources to hire social media content consultants to manage or support this aspect of program advertisement”. As the tips in the paper would of most value to those who are relative novices, these novices may be tempted to dive in, and be burnt as so many have before. Having a consultant would help to prevent that. In the absence of a consultant, a further tip may be added: proceed slowly and cautiously, and be prepared to delete and apologise when necessary. And it will be necessary.Still, a useful read for medical schools considering using social media for the resident (and other) recruitment.

Reviewer Expertise:

NA

No decision status is available
